# Reporting and Interpreting Task Performance in Go/No-Go Affective Shifting Tasks

**DOI:** 10.3389/fpsyg.2017.00701

**Published:** 2017-05-09

**Authors:** Adrian Meule

**Affiliations:** ^1^Department of Psychology, University of SalzburgSalzburg, Austria; ^2^Center for Cognitive Neuroscience, University of SalzburgSalzburg, Austria

**Keywords:** motor response inhibition, inhibitory control, behavioral inhibition, impulsivity, go/no-go, food cues

There is an increased interest in the study of impulsive reactions to food cues (Bartholdy et al., 2016). For this, computerized tasks that include lexical or pictorial food stimuli are often used. One of such tasks is the affective shifting task, which is a type of go/no-go task. Unfortunately, it appears that reporting and interpreting performance in this task has been fairly inconsistent across studies. Therefore, the current article aims to highlight some of these issues in an effort to provide some guidance for researchers who are interested in using this task.

Response inhibition or inhibitory control is an executive function, which involves controlling one's attention, behavior, thoughts, and/or emotions to override a strong internal predisposition or external lure (Diamond, 2013). Prominent psychological tasks that are used to measure inhibitory control include, for example, the Stroop task, Simon task, Flanker task, antisaccade tasks, or stop-signal tasks (Diamond, 2013). Another widely used measure is go/no-go tasks. In these tasks, participants are usually required to perform a quick motor response (e.g., pressing a button on a keyboard as fast as possible) when certain stimuli (i.e., targets) are displayed on a computer screen and to withhold this reaction for other stimuli (i.e., non-targets; also called distractors or lures). As this is an easy task, go/no-go paradigms usually involve a large number of trials of which only a small number of trials are no-go trials (e.g., Kaufman et al., 2003). This is necessary in order for the go-reaction to become pre-potent and, thus, to ensure that participants make enough commission errors (i.e., falsely pressing the button in no-go trials; also called false alarms). Reaction times in or the number of correct go-trials (i.e., hits) and omission errors (i.e., falsely not pressing the button in go trials; also called misses) can also be calculated, but they are usually not considered as indices of inhibitory control (or, specifically, lack thereof).

Murphy et al. (1999) developed a modified version of such go/no-go tasks, which they termed *affective shifting task*. In their task, words were presented on the screen one by one. Participants had to respond to targets by pressing the space bar as quickly as possible but withhold responses to distractors. The task comprised two practice blocks followed by eight test blocks, each including nine happy words and nine sad words (presented in randomized order within each block). Before each block, either happy or sad words were specified as targets. This means that when participants had to press the button in response to happy words, they were required to not press the button in response to sad words (or vice versa). The 10 blocks were presented either in the order happy-happy-sad-sad-happy-happy-sad-sad-happy-happy or sad-sad-happy-happy-sad-sad-happy-happy-sad-sad (with happy or sad indicating the respective target here). Thus, four test blocks were *shift* blocks, for which the instruction was reversed as compared to the previous block and four blocks were *non-shift* blocks, for which the instruction was the same as in the previous block.

It might be argued that the arrangement of the affective shifting task demands mental flexibility and, thus, it may not be a pure measure of inhibitory control. Although this might be a potential confound, the task also has a practical advantage over traditional go/no-go tasks. The instruction shifts increase task difficulty and, thus, a lower number of trials is sufficient for producing enough commission errors. Specifically, as opposed to traditional go/no-go tasks, there is an equal number of go and no-go trials and a low number of trials in total. Consider, for example, that the task employed by Murphy et al. (1999) merely contained 180 trials while other go/no-go tasks contain more than 1,000 trials (e.g., X-Y task, see Garavan et al., 2002; Kaufman et al., 2003; Meule et al., 2011). In addition, the several shifts make the single blocks very short und allow participants to take a break. Thus, the task is more comfortable (e.g., less boring) for participants and, thus, motivation may be higher to perform the task correctly than when using more exhausting go/no-go tasks.

Murphy et al. (1999) and others used the affective shifting task with emotional words (Rubinsztein et al., 2000; García-Blanco et al., 2013) or emotional pictures (Lange et al., 2012). However, the task can easily be adapted to other lexical or pictorial stimuli. For example, others have used versions with alcohol-related words (Noël et al., 2005, 2007) or pictures (Adams et al., 2013; Czapla et al., 2016) and food-related words (Mobbs et al., 2008, 2011; Loeber et al., 2012, 2013) or pictures (Meule and Kübler, 2014; Meule et al., 2014; Deux et al., 2017).

Unfortunately, there seems to be quite a confusion about which task performance indices should be reported and how these can be interpreted. Some studies have reported indices based on signal detection theory such as discrimination index *d'* or decision bias *C* and it has been argued that *C* is a better indicator of disinhibition than commission errors alone as it takes the number of both hits and false alarms into account (Noël et al., 2005; Mobbs et al., 2008). Yet, it is not clear if these should be actually preferred over simpler measures. For example, the same authors switched to reporting the more straightforward reaction times, omission errors and commission errors in their later works (Noël et al., 2007; Mobbs et al., 2011). While omission errors may indicate lapses of attention, reaction times have been interpreted as reflecting an “attention and/or response bias” (Murphy et al., 1999, p. 1314) or “an approach tendency” (Meule et al., 2014, p. 12). Yet, these interpretations lack a substantive empirical basis and are, therefore, speculative. While a high number of commission errors is widely accepted to index low inhibitory control (Schulz et al., 2007), interpretation is also not that trivial in the affective shifting task. As would be expected, non-shift blocks are easier than shift blocks and, accordingly, participants commit more errors in shift blocks than in non-shift blocks. However, when examining the seven studies that used food-related affective shifting tasks, only four of them (Mobbs et al., 2008, 2011; Meule and Kübler, 2014; Meule et al., 2014) reported results as a function of block type (i.e., shift vs. non-shift).

To illustrate the importance of block type, I have combined data from two studies, in which food-related affective shifting tasks and a short form of the Barratt Impulsiveness Scale (Spinella, 2007) were used in two samples of female students (Meule and Kübler, 2014; Meule et al., 2014, study 2). In shift blocks, commission errors (*M* = 11.4, *SD* = 4.76) were more common than in non-shift blocks (*M* = 7.12, *SD* = 3.85, *t*_(156)_ = 12.2, *p* < 0.001). As depicted in Figure 1, higher attentional impulsivity related to more commission errors in shift blocks [*r*_(*n* = 157)_ = 0.193, *p* = 0.015], but not in non-shift blocks [*r*_(*n* = 157)_ = 0.108, *p* = 0.178]. Higher motor impulsivity also related to more commission errors in shift blocks [*r*_(*n* = 157)_ = 0.264, *p* = 0.001], but not in non-shift blocks [*r*_(*n* = 157)_ = 0.138, *p* = 0.086]. Non-planning impulsivity neither correlated with commission errors in shift blocks [*r*_(*n* = 157)_ = 0.138, *p* = 0.086] nor in non-shift blocks [*r*_(*n* = 157)_ = 0.076, *p* = 0.347]. When examining the relationships between all three impulsivity subscales and the number of commission errors together in one linear regression analysis, only motor impulsivity (β = 0.208, *p* = 0.026) but not attentional (β = 0.126, *p* = 0.122) or non-planning impulsivity (β = −0.002, *p* = 0.982) predicted the number of commission errors. Therefore, it appears that the construct that researchers actually want to measure (i.e., motor response inhibition/impulsivity) can primarily be found in shift blocks. Future studies are needed, however, in which other behavioral measures (e.g., stop-signal task) are used in order to provide further support for validity of commission errors in shift blocks as an index of motor response inhibition. Nevertheless, by not considering block type in analyses and interpretation of the data, researchers might miss important information and, therefore, may overlook interesting findings.

**Figure 1:**
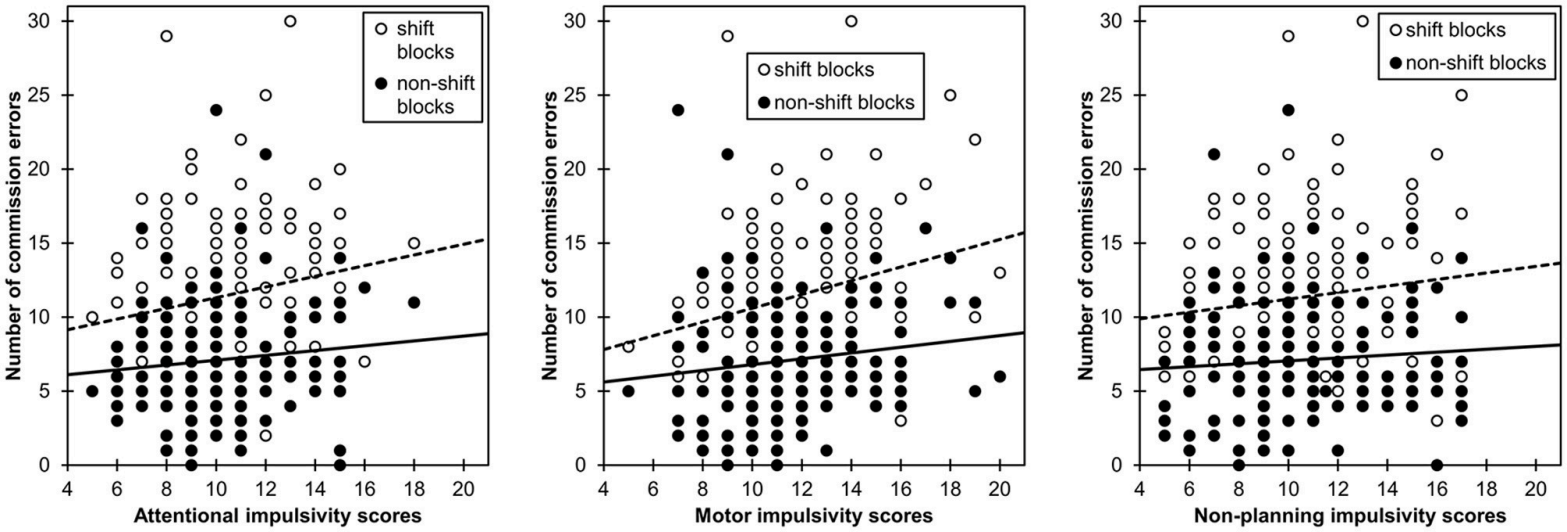
**Scatterplots illustrating relationships between the number of commission errors in a go/no-go affective shifting task and subscales scores of a short form of the Barratt Impulsiveness Scale as a function of block type (shift vs. non-shift blocks)**. Higher attentional and motor impulsivity scores related to a higher number of commission errors in shift blocks (dashed line), but not in non-shift blocks (solid line). Non-planning impulsivity scores were not correlated with the number of commission errors.

To complicate matters, researchers need to be aware that interpreting commission errors as a function of target type is different from reaction time and omission errors in this task. As an example, consider that a researcher uses an affective shifting task with food and neutral pictures. That is, participants have to either respond to food but not to neutral pictures in half of the blocks and to neutral but not to food pictures in the other half of the blocks. In food blocks (i.e., when food is the target category) reaction times represent reactions to food stimuli and omission errors represent missed reactions to food stimuli. Commission errors, however, represent false button presses in response to the distractors (i.e., neutral stimuli). While this may sound trivial, consider looking at the figures in Loeber et al. (2012) or Meule et al. (2014), where reaction times and commission errors are displayed in one figure with the conditions (i.e., target types) labeled as *food* and *neutral/objects*. As reaction time in food blocks refer to reactions to food, commission errors in food blocks refer to reactions to objects. This twist is a specific feature of the affective shifting task as the meaning of the go and no-go stimuli is either not reversed (e.g., Batterink et al., 2010) or separate blocks with only food and only neutral stimuli are used (e.g., Houben et al., 2014) in other response inhibition tasks (but also see Teslovich et al., 2014).

Related to this issue is the question of whether more errors would be expected in blocks with food targets (i.e., neutral distractors) or in blocks with neutral targets (i.e., food distractors). It could be argued that having to respond to appealing food stimuli will lead participants to accidently press the button in response to neutral distractors as well. However, it may also be that food distractors will lure them to commit more errors when they actually are supposed to respond to neutral stimuli only. This second hypothesis was confirmed in the studies by Loeber et al. (2012) and Meule et al. (2014; study 2): participants committed more errors when neutral stimuli were targets than when food stimuli were targets (i.e., they committed more errors in response to food distractors than to neutral distractors). However, this difference was only found in hungry participants in one study (Loeber et al., 2013), but was unrelated to current hunger in two other studies (Meule et al., 2014). Body weight did not interact with condition (i.e., food vs. neutral) regarding the number of commission errors (Mobbs et al., 2011; Loeber et al., 2012; Deux et al., 2017). When using low-calorie food stimuli instead of neutral stimuli as control category, participants who frequently experienced cravings for high-calorie foods (Meule and Kübler, 2014) and non-overweight adolescents (Deux et al., 2017) committed more errors to low-calorie food distractors (i.e., when high-calorie foods were targets) than to high-calorie food distractors (i.e., when low-calorie foods were targets). In conclusion, it is my contention that is currently unclear in which type of condition [i.e., (high-calorie) food category as targets vs. (low-calorie or neutral) control category as targets] higher or lower inhibitory performance can be expected and how potential moderators (e.g., current hunger or body weight) may influence this inhibitory performance as a function of condition.

In conclusion, writing this opinion piece was motivated by increased interest in and inconsistent reporting and interpretation of the affective shifting task as indicated by both the number of publications in recent years and personal requests that I received about this task. Because of its briefness and simplicity, I think that food-related affective shifting tasks represent a promising tool for the investigation of impulsive reactions toward food cues. Although versions of the task have already been employed in students (Loeber et al., 2013; Meule and Kübler, 2014; Meule et al., 2014), individuals with bulimia (Mobbs et al., 2008), obese adults (Mobbs et al., 2011; Loeber et al., 2012), and adolescent, psychiatric inpatients (Deux et al., 2017), it appears that different task designs and analyses lead to a rather confusing literature. Some of this confusion may be resolved by standardized reporting of task performance (e.g., reaction times, omission errors, and commission errors as a function of both block type and condition) and careful interpretation of this task performance (e.g., whether it is the distractors that lead participants to commit errors or whether it is the targets that lead participants to also respond to the distractors). I hope that this paper may provide a useful guidance for researchers who are interested in using this task, whether food-related or in other fields of research.

## Author contributions

The author confirms being the sole contributor of this work and approved it for publication.

### Conflict of interest statement

The author declares that the research was conducted in the absence of any commercial or financial relationships that could be construed as a potential conflict of interest.

## References

[B1] Adams S. Ataya A. F. Attwood A. S. Munafò M. R. (2013). Effects of alcohol on disinhibition towards alcohol-related cues. Drug Alcohol Depend. 127, 137–142. 10.1016/j.drugalcdep.2012.06.025 22841455

[B2] Bartholdy S. Dalton B. O'Daly O. G. Campbell I. C. Schmidt U. (2016). A systematic review of the relationship between eating, weight and inhibitory control using the stop signal task. Neurosci. Biobehav. Rev. 64, 35–62. 10.1016/j.neubiorev.2016.02.010 26900651

[B3] Batterink L. Yokum S. Stice E. (2010). Body mass correlates inversely with inhibitory control in response to food among adolescent girls: an fMRI study. Neuroimage 52, 1696–1703. 10.1016/j.neuroimage.2010.05.059 20510377 PMC2910204

[B4] Czapla M. Vollstädt-Klein S. Fauth-Bühler M. Best E. Fix M. Mann K. (2016). Response inhibition deficits: reliability of alcohol-related assessment tasks. Sucht 62, 203–215. 10.1024/0939-5911/a000431

[B5] Deux N. Schlarb A. A. Martin F. Holtmann M. Hebebrand J. Legenbauer T. (2017). Overweight in adolescent, psychiatric inpatients: A problem of general or food-specific impulsivity? Appetite 112, 157–166. 10.1016/j.appet.2017.01.031 28131756

[B6] Diamond A. (2013). Executive functions. Annu. Rev. Psychol. 64, 135–168. 10.1146/annurev-psych-113011-143750 23020641 PMC4084861

[B7] Garavan H. Ross T. J. Murphy K. Roche R. A. P. Stein E. A. (2002). Dissociable executive functions in the dynamic control of behavior: inhibition, error detection, and correction. Neuroimage 17, 1820–1829. 10.1006/nimg.2002.1326 12498755

[B8] García-Blanco A. C. Perea M. Livianos L. (2013). Mood-congruent bias and attention shifts in the different episodes of bipolar disorder. Cogn. Emot. 27, 1114–1121. 10.1080/02699931.2013.764281 23360445

[B9] Houben K. Nederkoorn C. Jansen A. (2014). Eating on impulse: the relation between overweight and food-specific inhibitory control. Obesity 22, E6–E8. 10.1002/oby.20670 24910860

[B10] Kaufman J. N. Ross T. J. Stein E. A. Garavan H. (2003). Cingulate hypoactivity in cocaine users during a GO–NOGO task as revealed by event-related functional magnetic resonance imaging. J. Neurosci. 23, 7839–7843. 12944513 10.1523/JNEUROSCI.23-21-07839.2003 PMC6740597

[B11] Lange C. Adli M. Zschucke E. Beyer R. Ising M. Uhr M.. (2012). Affective set-shifting deficits in patients with major depression in remission. J. Psychiatr. Res. 46, 1623–1626. 10.1016/j.jpsychires.2012.09.007 23040865

[B12] Loeber S. Grosshans M. Herpertz S. Kiefer F. Herpertz S. (2013). Hunger modulates behavioral disinhibition and attention allocation to food-associated cues in normal-weight controls. Appetite 71, 32–39. 10.1016/j.appet.2013.07.008 23899903

[B13] Loeber S. Grosshans M. Korucuoglu O. Vollmert C. Vollstädt-Klein S. Schneider S.. (2012). Impairment of inhibitory control in response to food-associated cues and attentional bias of obese participants and normal-weight controls. Int. J. Obes. 36, 1334–1339. 10.1038/ijo.2011.184 21986703

[B14] Meule A. Kübler A. (2014). Double trouble: trait food craving and impulsivity interactively predict food-cue affected behavioral inhibition. Appetite 79, 174–182. 10.1016/j.appet.2014.04.014 24768896

[B15] Meule A. Lukito S. Vögele C. Kübler A. (2011). Enhanced behavioral inhibition in restrained eaters. Eat. Behav. 12, 152–155. 10.1016/j.eatbeh.2011.01.006 21385646

[B16] Meule A. Lutz A. P. C. Krawietz V. Stützer J. Vögele C. Kübler A. (2014). Food-cue affected motor response inhibition and self-reported dieting success: a pictorial affective shifting task. Front. Psychol. 5:216. 10.3389/fpsyg.2014.00216 24659978 PMC3952046

[B17] Mobbs O. Iglesias K. Golay A. Van der Linden M. (2011). Cognitive deficits in obese persons with and without binge eating disorder. Investigation using a mental flexibility task. Appetite 57, 263–271. 10.1016/j.appet.2011.04.023 21600255

[B18] Mobbs O. Van der Linden M. d'Acremont M. Perroud A. (2008). Cognitive deficits and biases for food and body in bulimia: Investigation using an affective shifting task. Eat. Behav. 9, 455–461. 10.1016/j.eatbeh.2008.07.002 18928909

[B19] Murphy F. C. Sahakian B. J. Rubinsztein J. S. Michael A. Rogers R. D. Robbins T. W.. (1999). Emotional bias and inhibitory control processes in mania and depression. Psychol. Med. 29, 1307–1321. 10.1017/S0033291799001233 10616937

[B20] Noël X. Van der Linden M. d'Acremont M. Bechara A. Dan B. Hanak C.. (2007). Alcohol cues increase cognitive impulsivity in individuals with alcoholism. Psychopharmacology 192, 291–298. 10.1007/s00213-006-0695-6 17279375

[B21] Noël X. Van der Linden M. d'Acremont M. Colmant M. Hanak C. Pelc I.. (2005). Cognitive biases toward alcohol-related words and executive deficits in polysubstance abusers with alcoholism. Addiction 100, 1302–1309. 10.1111/j.1360-0443.2005.01125.x 16128719

[B22] Rubinsztein J. S. Michael A. Paykel E. S. Sahakian B. J. (2000). Cognitive impairment in remission in bipolar affective disorder. Psychol. Med. 30, 1025–1036. 10.1017/S0033291799002664 12027040

[B23] Schulz K. P. Fan J. Magidina O. Marks D. J. Hahn B. Halperin J. M. (2007). Does the emotional go/no-go task really measure behavioral inhibition? Convergence with measures on a non-emotional analog. Arch. Clin. Neuropsychol. 22, 151–160. 10.1016/j.acn.2006.12.001 17207962 PMC2562664

[B24] Spinella M. (2007). Normative data and a short form of the Barratt Impulsiveness Scale. Int. J. Neurosci. 117, 359–368. 10.1080/00207450600588881 17365120

[B25] Teslovich T. Freidl E. K. Kostro K. Weigel J. Davidow J. Y. Riddle M. C.. (2014). Probing behavioral responses to food: development of a food-specific go/no-go task. Psychiatry Res. 219, 166–170. 10.1016/j.psychres.2014.04.053 24909971 PMC4128315

